# Effects of Fenofibrate on Adiponectin Expression in Retinas of Streptozotocin-Induced Diabetic Rats

**DOI:** 10.1155/2014/540326

**Published:** 2014-12-01

**Authors:** Ying-Jung Hsu, Lu-Chun Wang, Wei-Shiung Yang, Chung-May Yang, Chang-Hao Yang

**Affiliations:** ^1^Graduate Institute of Clinical Medicine, College of Medicine, National Taiwan University, Taipei 100, Taiwan; ^2^Department of Ophthalmology, National Taiwan University Hospital, Taipei 100, Taiwan; ^3^Department of Internal Medicine, National Taiwan University Hospital, Taipei 100, Taiwan; ^4^Department of Ophthalmology, National Taiwan University Hospital, College of Medicine, National Taiwan University, No. 7 Chung-Shan South Road, Taipei 100, Taiwan

## Abstract

Adiponectin has been associated with increased risks of microvascular complications in diabetes; however, its role in the development of diabetic retinopathy (DR) is unknown. Fenofibrate is a lipid-lowering agent that has been shown to be capable of preventing DR progression. We investigated the expression of adiponectin and its receptors in DR and evaluated the effects of fenofibrate on their expression. The mRNA and protein levels of adiponectin and its receptors were elevated in retinas of streptozotocin-induced diabetic rats and were suppressed following fenofibrate treatment. Immunofluorescence staining demonstrated that adiponectin and adipoR1 were expressed in cells located within blood vessels, the retinal ganglion, and the inner nuclear layer. AdipoR1 was strongly expressed whereas adipoR2 was only weekly expressed in vascular endothelial cells. The* in vitro* experiments showed that adiponectin expression was induced by high glucose concentrations in RGC-5 and RAW264.7 cells and was suppressed following fenofibrate treatment. AdipoR1 and adipoR2 levels in RGC-5 cells were elevated in high glucose concentrations and suppressed by fenofibrate. Our results demonstrated that adiponectin may be a proinflammatory mediator in diabetic retinas and fenofibrate appears to modulate the expression of adiponectin and its receptors in diabetic retinas, effectively reducing DR progression.

## 1. Introduction

Diabetic retinopathy (DR) affects approximately 150 million people worldwide and is the leading cause of vision loss in adults of working age in industrialized countries [1]. DR is the most common microvascular complication of diabetes [2], and retinal vascular leakage, inflammation, and neovascularization are its main features [3]. Previous clinical studies have revealed that increased vitreous and serum proinflammatory cytokines are correlated with DR progression [4, 5]. The associated inflammation induces retinal vessel occlusion, capillary degeneration, and eventually the formation of new vessels [6]. Because inflammation plays an important role in DR pathogenesis, anti-inflammatory agents may be valuable in the development of therapeutic treatments to ameliorate DR progression.

Adiponectin is an adipocyte-specific protein that is secreted by adipose cells and mimics many of the metabolic activities of insulin [7, 8]. Two adiponectin receptors have been identified, including adipoR1 and adipoR2, which are involved in activating 5′ adenosine monophosphate-activated protein kinase (AMPK) and peroxisome proliferator-activated receptor *α* (PPAR-*α*), respectively [9]. Adiponectin has been reported to play a protective role against diabetes and cardiovascular diseases [7, 8]. However, it is also associated with increased risks of diabetic microvascular complications, such as retinopathy and nephropathy [10], which may result from adiponectin-stimulated angiogenesis [11, 12]. Clinical research has demonstrated that increased serum adiponectin concentrations are correlated with DR [13, 14]. Furthermore, aqueous humor (AqH) adiponectin levels have also been shown to be significantly higher in proliferative diabetic retinopathy (PDR) patients compared with control patients [15, 16]. Previous research has also shown that adiponectin and its receptors exist in type 1 diabetic human and mouse retinas [17]. However, the role of adiponectin and its receptors in DR still remains unknown.

Fenofibrate is a lipid-lowering agent that is used to treat lipid abnormalities in patients who are at high risk for cardiovascular diseases [18]. Recently, two major clinical trials, including the Fenofibrate Intervention and Event Lowering in Diabetes [19] study and the Action to Control Cardiovascular Risk in Diabetes- (ACCORD-) Eye Study, showed that fenofibrate reduced the progression of DR, and this activity was not associated with its lipid-lowering effects [19, 20]. Fenofibrate stimulates PPAR-*α* and modulates the AMPK pathway [21, 22] which is involved in inflammation, oxidative stress, and vascular responses. Therefore, it may be beneficial in the treatment of DR due to its anti-inflammatory and antioxidative effects and improved vascular reactivity [19], although the underlying mechanisms remain to be elucidated.

In previous studies, fenofibrate increased plasma adiponectin in patients with hypertriglyceridemia [23, 24] and suppressed adipoR1 protein levels in HepG2 cells (a hepatocellular carcinoma cell line) [25]. Additionally, fenofibrate treatment restored adipoR2 expression by reducing endoplasmic reticulum (ER) stress and inflammatory proteins in human hepatoma cells [26]. The effects of fenofibrate on the expression of adiponectin and its receptors require further investigation.

The present study had two main objectives. The first was to clarify the role of adiponectin and its receptors in DR pathogenesis, and the second was to investigate the effects of fenofibrate on the expression of adiponectin and its receptors in DR. We examined the effects of fenofibrate in streptozotocin- (STZ-) induced diabetic rats* in vivo* and in high glucose-stimulated cell lines, including those of retinal neuron cells, retinal vascular endothelial cells, and mouse macrophage cells* in vitro*.

## 2. Materials and Methods

### 2.1. Streptozotocin-Induced Diabetic Rats

Female 6- to 8-week-old Sprague Dawley (SD) rats weighing 220 to 250 g (*n* = 40, supplied by Animal Resource Center, College of Medicine, National Taiwan University) were used for the experiments. All of the animals were treated according to a protocol that was approved by the Institutional Animal Care and Use Committee of National Taiwan University in accordance with The Association for Research in Vision and Ophthalmology (ARVO) Statement for the use of animals in ophthalmic and vision research. The rats were randomly divided into four groups, including a normal control (C group, *n* = 10), diabetic rats (DM group, *n* = 10), diabetic rats that were treated with 30 mg/kg/day fenofibrate (DM+FL group, *n* = 10), and diabetic rats that were treated with 100 mg/kg/day fenofibrate (DM+FH group, *n* = 10). The rats in the diabetes and fenofibrate-treatment groups were administered an intraperitoneal injection of STZ (Sigma-Aldrich Co.), 60 mg/kg, dissolved in citrate buffer [pH 4.5] (Sigma-Aldrich Co.) to induce diabetes, and the control group received a sham injection of a similar volume of citrate buffer (pH 4.5). Three days after the STZ injections, blood glucose levels reached 250 mg/dL, indicating the successful induction of diabetes. Over the following 2 months, the rats in the treatment groups were administered 30 mg/kg or 100 mg/kg micronized fenofibrate (Laboratories Fournier S.A., Dijon, France) daily via an intragastric feeding tube. The diabetes group received intragastric feedings of normal saline in comparable amounts. The control group did not receive any intervention. Regular diets were supplied for all of the rats that did not receive insulin injections. Body weights and blood glucose levels were recorded at the beginning and end of the experiment. Serum total cholesterol levels were measured at the end of the experiments using Autoanalyzer 7070 (Hitachi Ltd.).

### 2.2. Tissue Preparation

The rats were anesthetized with a lethal dose of pentobarbital by intraperitoneal injection 2 months after diabetes inductions. The rats' abdominal cavities were opened, and plasma was collected by direct puncture of the descending aorta. The eyes were rapidly harvested and dissected. The retinas were carefully isolated under a microscope and stored at −80°C. The aqueous humor (AqH) was also collected for further investigations.

### 2.3. Cell Culture

RAW264.7 cells and RF/6A cells were grown in Dulbecco's modified Eagle's medium (DMEM) included 4.5 g/L D-glucose and supplemented with 10% fetal bovine serum (FBS), 100 *μ*g/mL streptomycin, and 100 units/mL penicillin (all from Invitrogen-Gibco, Carlsbad, CA, USA). RGC-5 cells were grown in Roswell Park Memorial Institute medium (RPMI-1640) included 2 g/L D-glucose and supplemented with 100 *μ*g/mL streptomycin, 100 units/mL penicillin, and 1% nonessential amino acids (NEAA) (all from Invitrogen-Gibco, Carlsbad, CA, USA). All cells were maintained at 37°C in a humidified, 5% CO_2_ environment.

### 2.4. Cell Experimental Design

RAW264.7 cells were exposed to 0, 5, 10, 15, and 25 mM glucose for 24 hours (acute condition). RF/6A and RGC-5 cells were exposed to 0, 5, 10, 20, and 30 mM glucose for 24 hours. The maximal effects were obtained at 25 mM glucose for the RAW264.7 cells and at 30 mM glucose for the RGC-5 and RF/6A cells; therefore, the mechanisms that are involved in chemokine modulation were examined in cells exposed to 25 mM and 30 mM glucose; a normal glucose concentration (0 mM) was used as the control. Prior to the fenofibrate treatment, the cells were incubated with 10 *μ*M GW6471 (a PPAR-*α* antagonist, R&D systems, Minneapolis, MN, USA) for 1 hour. The cells were pretreated with 50 *μ*M or 100 *μ*M fenofibrate for 1 hour prior to the glucose treatment. After the 24-hour glucose treatment, the cells were collected and further analyzed.

### 2.5. Preparation of RNA and cDNA

Total RNA was extracted from the retinas using the TRIzol reagent (Invitrogen-Life Technologies Inc., Gaithersburg, MD). For each sample, 1 *μ*g of total RNA was incubated with 300 ng of Oligo dT (Promega, Madison, WI, USA) for 5 min at 65°C and reverse-transcribed into cDNA using 80 U of Moloney murine leukemia virus reverse transcriptase (MMLV-RT; Invitrogen-Gibco, Grand Island, NY, USA) per 50 *μ*g reaction sample for 1 hour at 37°C. The reaction was stopped by heating the samples for 5 min at 90°C.

### 2.6. Semiquantitative PCR

PCR was performed on the resultant cDNA from each sample using adiponectin, adipoR1, adipoR2, and *β*-actin primers. All of the primers were prepared by Mission Biotech (Taipei, Taiwan). The amplification was performed using a thermocycler (MJ Research, Waltham, MA, USA). The 25 *μ*L reaction mixture consisted of 5 *μ*L of cDNA, 1 *μ*L of sense and antisense primers, 200 *μ*M of each deoxynucleotide (DTT), 5 *μ*L of 10x Taq polymerase buffer, and 1.25 U of GoTaq polymerase (Promega, Midison, WI, USA). The PCR reaction was performed using an annealing temperature of 56°C with GoTaq polymerase, cDNA, and the following primers: sense 5′-AATCCTGCCCAGTCATGAAG and antisense 5′-GGAACATTGGGGACAGTGC for adiponectin; sense 5′-AGACCACCTATGCCCTCCTT and antisense 5′-GCTGTGGGGAGCAGTAGAAG for adipoR1; sense 5′-TGGGAAGTTTTGTTCCTTGG and antisense 5′-TAGAGGGCAGCTCCTGTGAT for adipoR2; and sense 5′-CTGGAGAAGAGCTATGAGCTG and antisense 5′-AATCTCCTTCTGCATCCTGTC for *β*-actin. The DNA fragments were amplified for 25–30 cycles (30 sec at 94°C; 1 min at 50–52°C; 1 min at 72°C) followed by a final 7 min extension step at 72°C. The products were subjected to electrophoresis on a 1.5% agarose gel and analyzed using a gel analyzer system. Each mRNA level was normalized relative to the *β*-actin mRNA levels.

### 2.7. Protein Extractions and Western Blot Analysis

The proteins were extracted from the retinal homogenates or cells using radioimmunoprecipitation assay (RIPA) lysis buffer, which contained 0.5 M Tris-HCl (pH 7.4), 1.5 M NaCl, 2.5% deoxycholic acid, 10% NP-40, 10 mM EDTA, and 10% protease inhibitors (Complete Mini; Roche Diagnostics Corp., Indianapolis, IN, USA). For the western blot analysis, the protein samples were separated using a 10% sodium dodecyl sulfate- (SDS-) polyacrylamide gel and transferred to a polyvinylidene difluoride membrane (Immobilon-P; Millipore Corp., Billerica, MA, USA). The analysis was performed using anti-adiponectin (Cell Signaling Technology Inc. for the rat and cell samples at a 1 : 1000 dilution), anti-adiponectin receptor 1 (Santa Cruz Biotechnology Inc., for the rat samples at a 1 : 500 dilution; Epitomics, Inc., Burlingame, CA, USA, for the cell samples at a 1 : 500 dilution), anti-adiponectin receptor 2 (Santa Cruz Biotechnology Inc., for the rat samples at a 1 : 500 dilution; Bioss Inc., Woburn, MA, USA, for the cell samples at a 1 : 2000 dilution), or anti-*β*-actin antibodies (Bioss Inc., Woburn, MA, USA, for all of the samples at a 1 : 5000 dilution). Immunodetection was performed by enhanced chemiluminescence (Pierce Biotechnology, Rockford, IL, USA) according to the manufacturer's instructions. Protein levels were determined using densitometry analysis of the protein bands.

### 2.8. Quantification of Adiponectin, Monocyte Chemoattractant Protein-1, and Interleukin-8 in Aqueous and Plasma 

The levels of adiponectin, monocyte chemoattractant protein-1 (MCP-1), and interleukin-8 (IL-8) in the aqueous and plasma of rats were quantified using sandwich enzyme-linked immunosorbent assay (ELISA) kits according to the manufacturers' instructions. The levels of adiponectin (Assaypro LLC.), MCP-1 (RayBiotech Inc., Norcross, GA, USA), and IL-8 (Uscn Life Science Inc., Wuhan, China) in the plasma samples that were obtained from the same rats were also measured. The ELISA was repeated 3 times. The samples were diluted up to 50 *μ*L or 100 *μ*L for the tests. Optical density measurements were determined at A450 (absorbance at 450 nm) using a microplate reader (Bio-Rad Laboratories Inc.). The concentrations were determined from standard curves using recombinant standards that were supplied by the manufacturers.

### 2.9. Immunofluorescence Staining of Adiponectin, AdipoR1, and AdipoR2

Formalin-fixed, 5 *μ*m, paraffin-embedded rat eye tissue sections were placed on slides, deparaffinized in xylenes, and rehydrated through graded ethanol into phosphate-buffered saline (PBS). Endogenous peroxidase was blocked using 0.3% hydrogen peroxide in methanol. Then, the sections were treated with 5% normal rat serum and incubated overnight with antibodies at 4°C. The following antibodies were used: rabbit polyclonal anti-adiponectin antibody (1 : 100 dilution; R&D Systems, Minneapolis, MN, USA), goat anti-adiponectin receptor 1 antibody (1 : 100 dilution; Phoenix Pharmaceuticals, Belmont, CA, USA), and goat anti-adiponectin receptor 2 antibody (1 : 100 dilution; Phoenix Pharmaceuticals Inc., Burlingame, CA, USA). Thereafter, the secondary goat anti-rabbit IgG-FITC antibody was added to PBS containing 1% BSA and incubated with the slides for 80 min in the dark. Finally, the slides were washed in PBS 5 times and mounted using mounting medium containing DAPI (Vector Labratories Inc., Burlingame, CA, USA).

### 2.10. Statistical Analyses

The data that were obtained from the experiments were expressed as the mean ± SD. The Mann-Whitney *U* test was used for the statistical evaluations. *P* values < 0.05 were considered to be statistically significant.

## 3. Results

### 3.1. Experimental Data for SD Rats

We measured the body weights and blood sugar levels of the experimental groups at 3 days and 8 weeks following STZ injection (Table 1). Initial body weights ranged from 274.0 ± 17.6 g to 280 ± 13.7 g. Eight weeks after the STZ injections, the average body weight of the control group increased to 459.4 ± 30.3 g. The body weights of the DM, DM+FL, and DM+FH groups were significantly lower than that of the control group (*P* < 0.001 for all of the paired comparisons). The initial blood sugar levels of the STZ-induced diabetic groups (525.8 ± 83.5 mg/dL to 540.8 ± 76.3 mg/dL) were significantly higher than those of the control group (96.7 ± 21.1 mg/dL, *P* < 0.001 for all of the paired comparisons). Eight weeks after the STZ injections, the blood sugar levels of the STZ-induced diabetic groups (512.3 ± 70.7 mg/dL to 551.7 ± 56.3 mg/dL) were also significantly higher than those of the control group (170.7 ± 40.5 mg/dL, *P* < 0.001 for all of the paired comparisons) but did not significantly differ when compared with the STZ-induced diabetic groups at 3 days after induction. The fenofibrate treatment groups did not demonstrate decreased blood sugar levels. The serum total cholesterol levels of the DM group (104.2 ± 22.7 mg/dL) were significantly higher than the control group (55.4 ± 8.1 mg/dL, *P* < 0.001). The cholesterol levels of fenofibrate treatment groups were significantly decreased compared to DM group (DM+FL group: 73.2 ± 5.8 mg/dL, *P* = 0.009; DM+FH group: 63.6 ± 4.7 mg/dL, *P* = 0.002).

### 3.2. Effects of Fenofibrate on Adiponectin, AdipoR1, and AdipoR2 mRNA Levels in Rat Retinas

**Figure 1:**
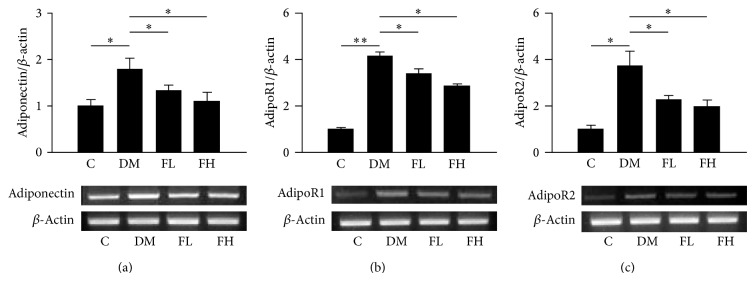
The evaluation of mRNA levels of adiponectin, adipoR1, and adipoR2 in rat retinas by semiquantitative PCR. The mRNA levels of adiponectin, adipoR1, and adipoR2 increased in retinas of the DM group. Fenofibrate decreased the levels of adiponectin, adipoR1, and adipoR2 relative to the levels observed in the DM group. The *y*-axis represents the ratios of adiponectin, adipoR1, and adipoR2 mRNA to *β*-actin mRNA in each group. The sample was pooled from one eye of five rats in each group. The data are expressed as the mean ± SD of three independent experiments (bar graph). ^*^ *P* < 0.05, ^**^ *P* < 0.001; DM group versus control group, fenofibrate groups versus DM group (FL: low-dose fenofibrate; FH: high-dose fenofibrate).

The mRNA levels of adiponectin, adipoR1, and adipoR2 were determined using semiquantitative PCR analysis (Figure 1). Compared with the control group, the adiponectin, adipoR1, and adipoR2 mRNA levels were significantly higher (*P* < 0.05 for adiponectin and adipoR2 and *P* < 0.001 for adipoR1) in the diabetic group. Treatment with fenofibrate significantly reduced the adiponectin, adipoR1, and adipoR2 mRNA levels (*P* < 0.001 for all of the paired comparisons) in the treated groups compared with the nontreated diabetic group. The mRNA levels of the high-dose fenofibrate group were not significantly lower than those of the low-dose group.

### 3.3. Effects of Fenofibrate on Adiponectin, AdipoR1, and AdipoR2 Protein Expression Levels in Rat Retinas

**Figure 2:**
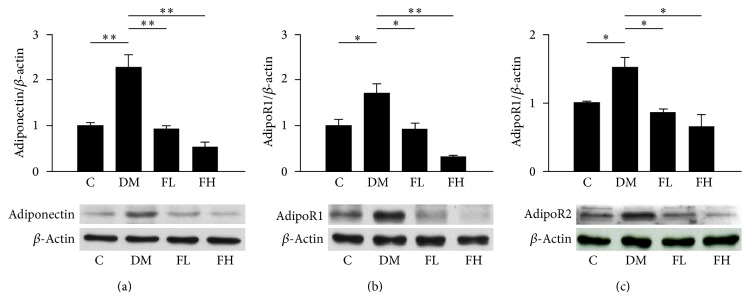
The evaluation of protein expression levels of adiponectin, adipoR1, and adipoR2 in rat retinas by western blot analysis. The protein expression levels of adiponectin and its receptors increased in the retinas of the DM group. Fenofibrate decreased the protein expression levels of adiponectin, adipoR1, and adipoR2 relative to the expression levels detected in the DM group. The *y*-axis represents the ratios of the adiponectin, adipoR1, and adipoR2 blot densities to the *β*-actin blot density in each group. The sample was pooled from one eye of five rats in each group. The data are expressed as the mean ± SD of three independent experiments (bar graph). ^*^ *P* < 0.05, ^**^ *P* < 0.001; DM group versus control group, fenofibrate groups versus DM group (FL: low-dose fenofibrate; FH: high-dose fenofibrate).

Western blot analysis was used to determine the protein expression levels of adiponectin, adipoR1, and adipoR2 in rat retinas (Figure 2). Compared with the control group, adiponectin, adipoR1, and adipoR2 showed significantly increased protein expression levels in the diabetes group (*P* < 0.05 for adipoR1 and adipoR2 and *P* < 0.001 for adiponectin). Treatment with fenofibrate significantly reduced the expression of adiponectin (*P* < 0.001 for all of the paired comparisons), adipoR1 (*P* < 0.05 for the FL group and *P* < 0.001 for the FH group), and adipoR2 (*P* < 0.05 for all of the paired comparisons) compared with the same expression levels that were observed in the rat retinas of the diabetes group. There were no significant differences between the high-dose and low-dose fenofibrate groups.

### 3.4. Effects of Fenofibrate on Adiponectin Concentration in Aqueous and Plasma

**Figure 3:**
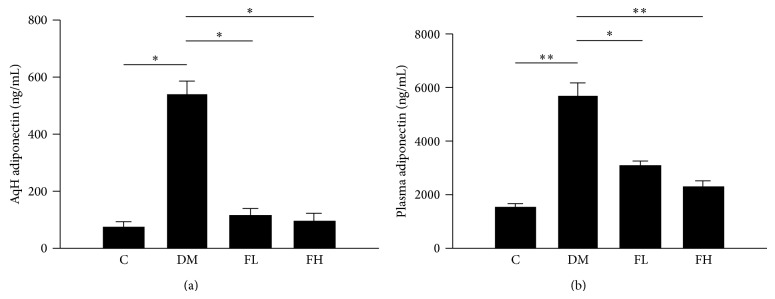
Quantification of the adiponectin levels in rat AqH (a) and plasma (b) using an ELISA. The adiponectin concentrations increased in the AqH and plasma of the DM group. Fenofibrate decreased the concentrations of adiponectin relative to the levels that were observed in the DM group. AqH was pooled from one eye of five rats in each group. The data are expressed as the mean ± SD of three independent experiments (bar graph). ^*^ *P* < 0.05, ^**^ *P* < 0.001; DM group versus control group, fenofibrate groups versus DM group (FL: low-dose fenofibrate; FH: high-dose fenofibrate).

The ocular and systemic adiponectin concentration was evaluated using an ELISA (Figure 3). The aqueous adiponectin concentration was significantly higher in the diabetic group compared with the control group (*P* < 0.001). Treatment with fenofibrate significantly lowered the aqueous adiponectin concentration in the diabetic group (low and high doses were *P* < 0.001) (Figure 3(a)). The plasma adiponectin concentration was also significantly higher in the diabetic group compared with the control group (*P* < 0.001). Treatment with fenofibrate significantly lowered the plasma adiponectin concentration (*P* < 0.05 for low doses and *P* < 0.001 for high doses) (Figure 3(b)). There were no significant differences between the high-dose and low-dose fenofibrate groups.

### 3.5. Effects of Fenofibrate on MCP-1 and IL-8 Concentrations in AqH and Plasma

**Figure 4:**
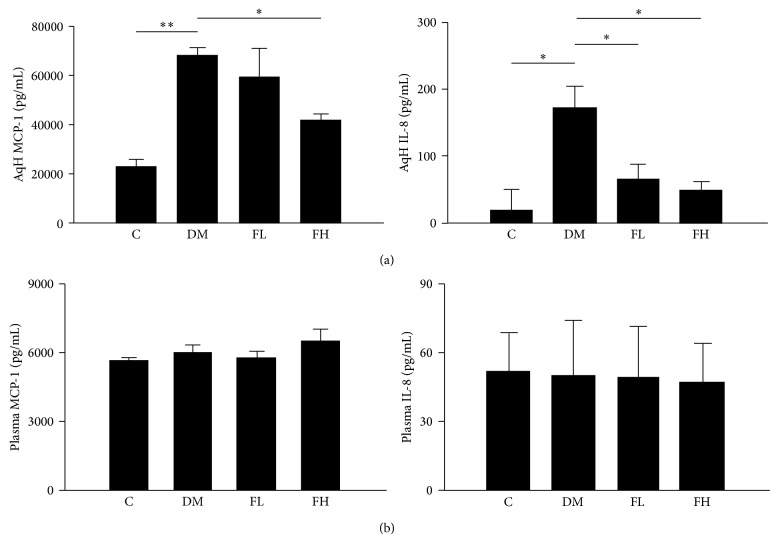
Quantification of MCP-1 and IL-8 levels in rat AqH (a) and plasma (b). MCP-1 and IL-8 concentrations increased in the AqH and plasma of the DM group. The low dose of fenofibrate decreased the concentrations of IL-8; the high dose decreased the concentration of MCP-1 and IL-8 relative to the levels in the DM group in the AqH but not the plasma. AqH was pooled from one eye of five rats, and plasma was collected from the abdominal aortas of individual rats in each group. The data are expressed as the mean ± SD of three independent experiments (bar graph). ^*^ *P* < 0.05, ^**^ *P* < 0.001; DM group versus control group, fenofibrate groups versus DM group (FL: low-dose fenofibrate; FH: high-dose fenofibrate).

The ocular and systemic MCP-1 and IL-8 concentrations in the experimental groups were evaluated using an ELISA (Figure 4). The AqH MCP-1 and IL-8 concentrations were significantly higher in the diabetic group compared with the control group (*P* < 0.001 for all of the paired comparisons). Treatment with fenofibrate significantly lowered the AqH IL-8 concentration in the diabetic group (*P* < 0.05 for all of the paired comparisons). Only the high-dose treatment reduced the MCP-1 concentration (*P* < 0.05) (Figure 4(a)). The plasma MCP-1 and IL-8 concentrations did not significantly differ among any of the groups (Figure 4(b)).

### 3.6. IF of Adiponectin, AdipoR1, and AdipoR2 in Retinas

**Figure 5:**
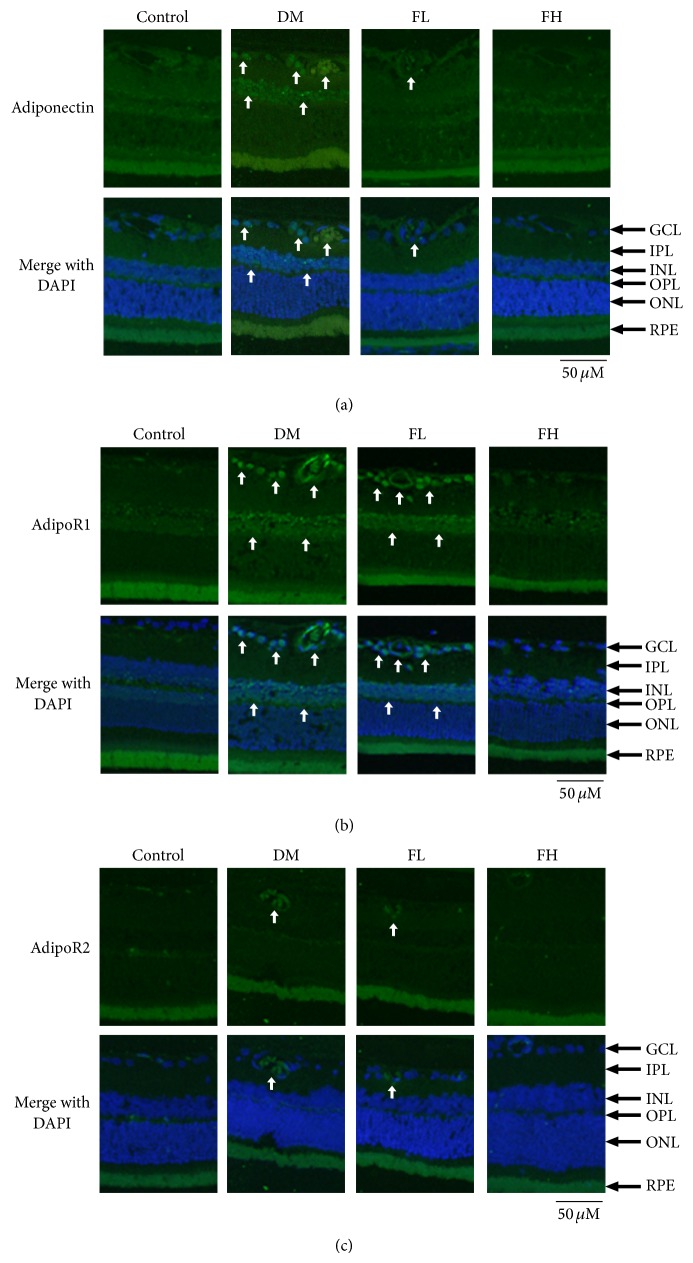
Effects of fenofibrate on DM rat retinas as visualized by immunofluorescence staining of adiponectin (a), adipoR1 (b), and adipoR2 (c). Adiponectin expression was observed in the cells within the blood vessels and ganglion cell layer (a). AdipoR1 was observed in the vascular endothelial cells and ganglion cell layer (b). AdipoR2 was expressed at low levels in the vascular endothelial cells (c). Positively stained cells are indicated by white arrows, and the original magnification is 200x (FL: low-dose fenofibrate; FH: high-dose fenofibrate; GCL: ganglion cell layer; IPL: inner plexiform; INL: inner nuclear layer; OPL: outer plexiform layer; ONL: outer nuclear layer; RPE: retinal pigment epithelium).

IF was performed to investigate the localization of adiponectin and its receptors in retinas (Figure 5). The strong expression of adiponectin and adipoR1 was detected in cells within the blood vessels, retinal ganglion cell layer, and inner nuclear layer in the diabetic group. Strong staining for adipoR1 was also observed in the retinal vascular cells. Low levels of adipoR2 expression were observed in the vascular endothelial cells in the diabetic group. In the fenofibrate treatment groups, decreased adiponectin and adipoR1 staining were observed compared with the diabetic group. There was no significant staining in the control group.

### 3.7. Effects of Fenofibrate on Glucose-Stimulated RGC-5 Cells

**Figure 6:**
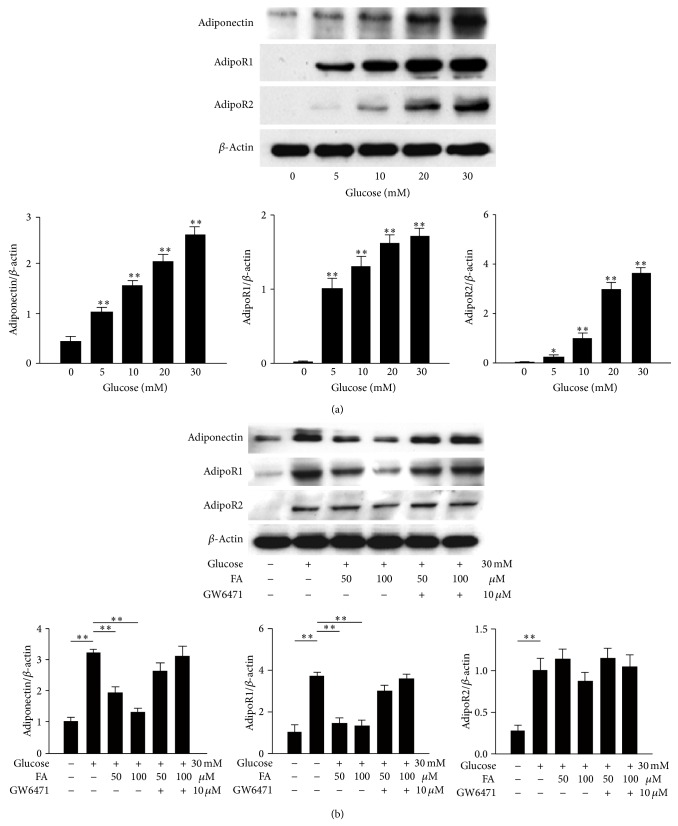
Evaluation of the protein expression levels of adiponectin, adipoR1, and adipoR2 in glucose-stimulated RGC-5 cells by western blot analysis. The protein expression levels increased with increasing glucose concentrations (a). Fenofibrate treatment decreased the expression levels of adiponectin, adipoR1, and adipoR2 relative to the expression levels observed in the DM group (b). The *y*-axis represents the ratios of the adiponectin, adipoR1, and adipoR2 blot densities to the *β*-actin blot density in each group. The sample was pooled from one eye of five rats in each group. The data are expressed as the mean ± SD of three independent experiments (bar graph). ^*^ *P* < 0.05, ^**^ *P* < 0.001; DM group versus control group, fenofibrate groups versus DM group.

Western blot analysis was used to analyze the protein levels of adiponectin, adipoR1, and adipoR2 in RGC-5 cells (Figure 6). Different concentrations of glucose stimulation led to increases in adiponectin, adipoR1, and adipoR2 expression in a dose-dependent manner. We selected a glucose concentration of 30 mM for further investigations (Figure 6(a)). Following pretreatment with fenofibrate, the levels of adiponectin and adipoR1 were significantly decreased (*P* < 0.001 for all of the paired comparisons) compared with cells treated with glucose only; additionally, no significant differences were detected between the two fenofibrate doses. The effects of fenofibrate on adiponectin and adipoR1 expression were blocked when the cells were pretreated with the PPAR-*α* antagonist GW6471. AdipoR2 levels did not significantly differ after the fenofibrate treatment (Figure 6(b)).

### 3.8. Effects of Fenofibrate on Glucose-Stimulated RAW264.7 Cells

**Figure 7:**
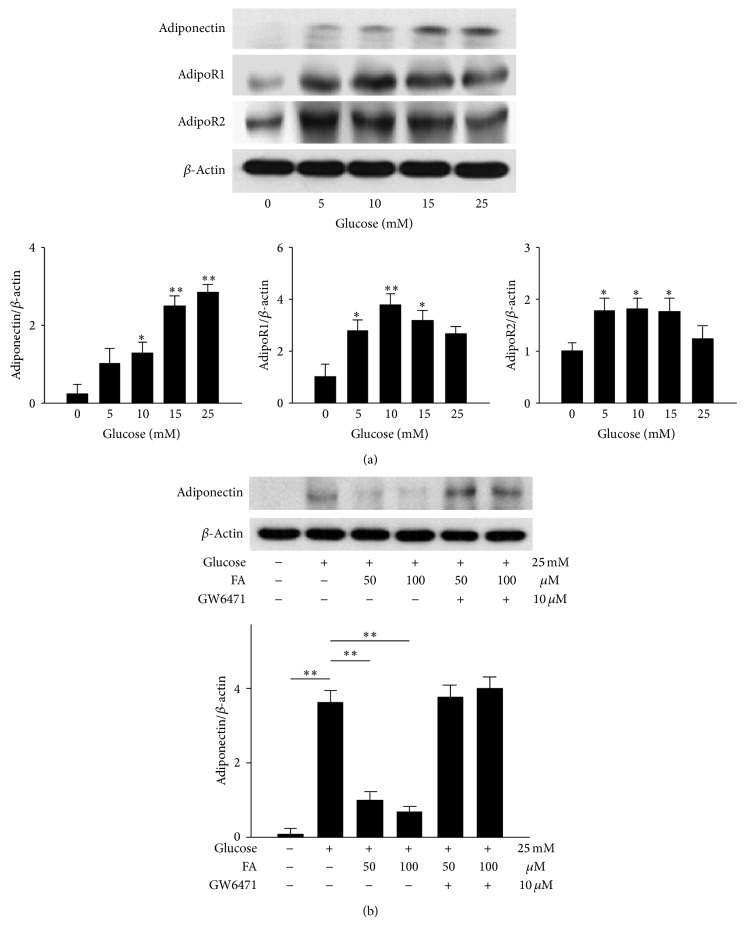
Evaluation of the protein expression levels of adiponectin, adipoR1, and adipoR2 in glucose-stimulated RAW264.7 cells by western blot analysis. Only adiponectin increased in a dose-dependent manner with increasing glucose concentrations (a). Fenofibrate decreased the expression of adiponectin relative to the expression levels observed in the DM group (b). The *y*-axis represents the ratios of the adiponectin, adipoR1, and adipoR2 blot densities to the *β*-actin blot density in each group. The sample was pooled from one eye of five rats in each group. The data are expressed as the mean ± SD of three independent experiments (bar graph). ^*^ *P* < 0.05, ^**^ *P* < 0.001; DM group versus control group, fenofibrate groups versus DM group.

Western blot analysis was used to analyze the protein expression levels of adiponectin, adipoR1, and adipoR2 in RAW264.7 cells (Figure 7). Adiponectin increased in a dose-dependent manner according to the glucose concentrations, from 10 to 25 mM. Although adipoR1 and adipoR2 were stimulated by 5 to 15 mM glucose, the expression of the receptors did not increase in a dose-dependent manner (Figure 7(a)). Pretreatment with fenofibrate resulted in significantly decreased adiponectin expression (*P* < 0.001 for both doses) compared with pretreatment with glucose only; no significant differences were observed between the two fenofibrate doses. The effects of fenofibrate on adiponectin were inhibited when the cells were pretreated with GW6471 (Figure 7(b)).

### 3.9. Effects of Fenofibrate on Glucose-Stimulated RF/6A Cells

**Figure 8:**
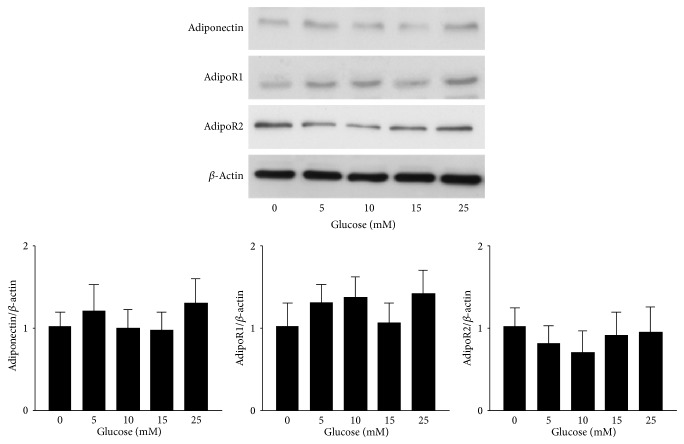
Evaluation of the expression levels of adiponectin, adipoR1, and adipoR2 in glucose-stimulated RF/6A cells by western blot analysis. The expression levels did not increase in a dose-dependent manner with increasing glucose concentrations. The *y*-axis represents the ratios of the adiponectin, adipoR1, and adipoR2 blot densities to the *β*-actin blot density in each group. The sample was pooled from one eye of five rats in each group. The data are expressed as the mean ± SD of three independent experiments (bar graph). ^*^ *P* < 0.05, ^**^ *P* < 0.001; DM group versus control group, fenofibrate groups versus DM group.

Western blot analysis was used to analyze the protein expression levels of adiponectin, adipoR1, and adipoR2 in RF/6A cells (Figure 8). No significant changes were observed following stimulation with different glucose concentrations.

## 4. Discussion

In the present* in vivo* study, we clearly demonstrated that there were significant increases in the mRNA and protein expression levels of adiponectin and its receptors in retinas of STZ-induced diabetic rats. The aqueous and plasma concentrations of adiponectin were also elevated. Daily treatment with fenofibrate inhibited these responses in retinas of the diabetic rats. IF staining revealed that adiponectin and adipoR1 were present in the cells within the blood vessels, the retinal ganglion cell layer, and the inner nuclear layer, and adipoR1 and adipoR2 were detected in the vascular endothelial cells in the diabetic group. We also investigated the effects of fenofibrate on high glucose-stimulated RGC-5, RAW264.7, and RF/6A cells. Adiponectin levels increased following stimulation with high concentrations of glucose and were suppressed by fenofibrate in RGC-5 and RAW264.7 cells. AdipoR1 and adipoR2 levels did not increase with increasing glucose concentrations in RAW264.7 and RF/6A cells. These results indicate that adiponectin is elevated under diabetic conditions, and fenofibrate may regulate the expression of adiponectin and its receptors under diabetic conditions. We did not have the data of control group treated with fenofibrate; however, there are two references describing that fenofibrate has no effect on adiponectin in normal condition. Castillero et al. showed that rat serum adiponectin concentration did not change significantly after fenofibrate treatment when it compared with group without fenofibrate treatment [27]. Gao et al. found that the mRNA level of adiponectin from adipose tissue of obese mice also did not change significantly whatever fenofibrate treatment or not [28]. So lack of control group treated with fenofibrate would not affect our experimental results.

In our animal study, we found that the expression of adiponectin and its receptors increased in diabetic rat retinas. Lin et al. demonstrated that the expression levels of adiponectin and adipoR1 were higher in type 1 diabetic eNOS knockout mice compared with control mice, which is consistent with our findings [17]. Adiponectin is typically considered to be a protective molecule with anti-inflammatory, antiatherosclerotic, and neuroprotective effects [29–31]. Adiponectin can hinder nuclear factor-*κ*B (NF-*κ*B) activation by attenuating proinflammatory cytokines [32] and suppress the vascular endothelial growth factor-induced migration of endothelial cells [33]. Therefore, the elevated expression of adiponectin in diabetes may represent a counterregulation to abate endothelial and vascular damage [34, 35] and mitigate the inflammatory effects of DR. However, adiponectin has also been reported to be a proinflammatory mediator. High adiponectin and adipoR1 expression levels were found in the synovial fluids and tissues of patients with rheumatoid arthritis [36], and adiponectin has also been shown to stimulate prostaglandin E_2_ (PGE_2_) production in the synovial fibroblasts of individuals with rheumatoid arthritis [37]. In type 1 diabetes patients, elevated adiponectin levels may mediate the induction of interleukin-6 (IL-6), MCP-1, and IL-8 [38]. Increased rates of IL-8 and MCP-1 production were detected in adiponectin-treated human microvascular endothelial cells and monocytes [39]. Taken together, these results indicate that adiponectin could play a dual role in the development of DR, serving as a counterregulatory agent and a proinflammatory mediator.

PPAR-*α* plays an important role in the regulation of fatty acid oxidation, lipid and lipoprotein metabolism, and vascular responses [40]. It also partially suppresses inflammation associated with the NF-*κ*B pathway, reduces oxidative stress damage, and inhibits angiogenesis [41]. Fenofibrate, which acts as a PPAR-*α* agonist, decreases several inflammatory mediators, including TNF-*α*, IL-6, and MCP-1 [42]. The FIELD study showed that fenofibrate reduced proliferative retinopathy by 30%, diminished its development and progression, and reduced the need for laser treatment in type 2 diabetic patients with preexisting DR in an ophthalmology substudy [19]. The ACCORD-Eye Study showed that a combination of fenofibrate and statin therapies also slowed DR progression under type 2 diabetic conditions [20]. In the present study, the MCP-1 and IL-8 concentrations in the aqueous and plasma were elevated in the diabetic rats. Treatment with fenofibrate reduced MCP-1 and IL-8 concentrations, which may be related to the anti-inflammatory effects of fenofibrate. We also demonstrated that fenofibrate suppresses the expression of adiponectin. Therefore, the anti-inflammatory activities that it exhibits in DR may be explained by its ability to suppress adiponectin, which typically acts as a proinflammatory mediator.

Aqueous humor and plasma adiponectin levels have been revealed to be higher in PDR patients with type 2 diabetes compared with non-DM control patients [13, 15]. In the present study, we found that adiponectin concentrations in the AqH and plasma were higher in the diabetic group than in the control group and were negatively correlated with body weight in the control and diabetic group at 8 weeks after STZ injection. Previous studies have demonstrated that serum adiponectin concentration is negatively correlated with body mass index (BMI) in obese individuals and type 2 diabetes patients [8, 43], which is consistent with our findings. The finding may be explained by the fact that DR typically causes vascular leakage due to disruptions in the blood-retinal barrier (BRB) [44]. Adiponectin may be transported through the compromised BRB into the aqueous by its receptors [17], than it could be released into the aqueous or bloodstream. Recently, research that was conducted by Chen et al. showed that fenofibrate attenuated retinal vascular permeability and reduced vascular leakage in type 1 diabetic rats [22]. In the present study, the increased concentrations of adiponectin in the aqueous and plasma may be related to the diabetes-induced vascular leakage. The protection against vascular damage that is conferred by fenofibrate may contribute to the reduced concentrations of adiponectin following fenofibrate treatment.

In the present study, IF staining was used to detect where adiponectin and its receptors were expressed within the retinas of diabetic rats. Adiponectin and adipoR1 expression were observed in the cells within blood vessels, retinal ganglion cells, and inner nuclear cells. Strong adipoR1 staining was also observed in vascular endothelial cells, whereas only light adipoR2 staining was observed in vascular endothelial cells. A previous study indicated that adipoR1 was present in the retinal pigment epithelia of human eyes, the photoreceptor outer segments in eNOS knockout mice, and the internal membranes of the retinas in human and mouse eyes [17]. Our findings contrasted with those from previous studies. These differences could have resulted from the different experimental methods. However, because there are few studies investigating the expression of adiponectin and its receptors in the retina, further research is necessary to clarify these results. Although adiponectin is known to be secreted from adipose tissue, adiponectin has also been reported to exist in renal tubular epithelial and aortic endothelial cells [45, 46]. Adiponectin receptors are expressed in prostate, gastric, breast, and endometrial cancer cells [47] and have also been found in human monocytic cells and colon epithelial cell [48, 49].

We also used high glucose-stimulated RGC-5, RAW264.7, and RF/6A cells, which represented retinal neuronal cells, mouse macrophage cells, and retinal vascular endothelial cells, respectively, to investigate the effects of fenofibrate on the expression of adiponectin and its receptors. When stimulated with glucose, the expression of adiponectin and its receptors increased in a dose-dependent manner in RGC-5 cells. Fenofibrate suppressed the expression of adiponectin and its receptors. The origin of RGC-5 cell line was disputed recently. It was proposed that RGC-5 processes similar properties with cone photoreceptor cell line 661W and perhaps a cross-contamination occurred in the origin [50]. We believed that it is still a useful tool for ophthalmological research. Therefore, in the present study, RGC-5 cell was used to represent the retinal neuronal cell. In RAW264.7 cells, adiponectin and its receptors were detected. However, only adiponectin responded to glucose stimulation in a dose-dependent manner, and fenofibrate treatment inhibited the expression of adiponectin. In RF/6A cells, the expression of adiponectin and its receptors was not affected by glucose stimulation. The diversity of these results may be due to the different sources of the cell lines, which showed varying levels of responsiveness to glucose stimulation.

Impaired liver function caused by fenofibrate has been reported in clinical studies [51–53]. Compared with the treatment dose in the FIELD study (200 mg daily in humans), 30 mg/kg/day [54] and 100 mg/kg/day [30, 55] fenofibrate were used in the present study. To apply this treatment clinically for the prevention of DR progression, more human studies and clinical trials are necessary to further delineate the safe dose and exact treatment guidelines for fenofibrate.

In conclusion, adiponectin and its receptors were elevated in diabetic rats, and fenofibrate treatment reduced their expression. Cell experiments revealed a diverse range of results in response to glucose stimulation that may be related to the different cell sources. The increases in the expression of adiponectin and its receptors in diabetic rats may play counterregulatory or proinflammatory roles in DR. The anti-inflammatory effects of fenofibrate could partially function by modulating the expression of adiponectin and its receptors, thus preventing DR progression.

## Figures and Tables

**Table 1:** The body weight and blood sugar of animal experimental groups.

Groups	Control	DM	DM+FL	DM+FH
3 days after STZ injection				
Body weight (g)	278.3 ± 8.3	277.0 ± 16.5	280.0 ± 3.7	274.0 ± 17.6
Blood sugar (mg/dL)	96.7 ± 21.1	525.8 ± 83.5^**^	526.6 ± 64.7^**^	540.8 ± 76.3^**^
8 weeks after STZ injection				
Body weight (g)	459.4 ± 30.3	323.0 ± 51.0^**^	350.5 ± 52.5^**^	347.9 ± 48.4^**^
Blood sugar (mg/dL)	170.7 ± 40.5	512.3 ± 70.7^**^	551.7 ± 56.3^**^	539.0 ± 75.0^**^
Serum TC (mg/dL)	55.4 ± 8.1	104.2 ± 22.7^**^	73.2 ± 5.8^*^	63.6 ± 4.7^*^

^**^ *P* < 0.001; ^*^ *P* < 0.05; DM group versus control group, fenofibrate group versus DM group (FL: fenofibrate low dose; FH: fenofibrate high dose; TC: total cholesterol); the blood sugar values were from fed rats.
